# Digital Analysis of Closest Speaking Space in Dentates—Method Proposal and Preliminary Findings

**DOI:** 10.3390/dj12110336

**Published:** 2024-10-22

**Authors:** Cristina Teodora Preoteasa, Karla Alexandra Duță, Bogdan Florin Tudose, Cătalina Murariu-Măgureanu, Elena Preoteasa

**Affiliations:** 1 Department of Scientific Research Methodology and Ergonomics, Faculty of Dental Medicine, “Carol Davila” University of Medicine and Pharmacy, 041313 Bucharest, Romania; 2 Faculty of Dental Medicine, “Carol Davila” University of Medicine and Pharmacy, 041313 Bucharest, Romania; karla.duta@stud.umfcd.ro; 3 3D Art Dental, 010464 Bucharest, Romania; 3dartdentallab@gmail.com; 4 Department of Dental Prosthodontics, Faculty of Dental Medicine, “Carol Davila” University of Medicine and Pharmacy, 041313 Bucharest, Romania; catalina.magureanu.murariu@umfcd.ro (C.M.-M.); elena.preoteasa@umfcd.ro (E.P.)

**Keywords:** functional space, mandibular movement, dental occlusion, freeway space, phonetic

## Abstract

Aims: The aim of this study is to identify methods for the digital analysis of the closest speaking space in dentates and to assess certain particularities using digital analysis. Method: For the adult patients included in this study, traditional dental casts were fabricated, and interocclusal registrations of the maximal intercuspal position and of the closest speaking space were taken using polyvinyl siloxane. Dental casts in both positions were scanned using a dental laboratory scanner, and digital analysis was conducted using the 3Shape 3D Viewer. Results: The interocclusal distance corresponding to the closest speaking space can be easily and precisely measured digitally or assessed using occlusion maps, at the level of all teeth. The interocclusal distance was variable across the dental arch, central incisors, and second molars, registering the smallest values, and was asymmetrical, with mandibular lateral deviation being suggested. The assessment of the range of motion of the mandible during the speaking test, recorded based on tooth movement, was conducted using superimpositions. The movement of the tooth was the largest in central incisors and decreased progressively as the tooth was more distal, and, in all cases, mandibular deviation occurred, more frequently to the left. Conclusions: Digital methods for the analysis of the closest speaking space have the advantages of increased precision and a broader range of analysis and application, showing research and clinical value.

## 1. Introduction

Dental treatment should be conducted so morphological and functional harmony is attained, promoting good short- and long-term prognosis. In this context, occlusion and a maxillomandibular relationship, e.g., with regard to occlusal contacts and the interocclusal distance, during mastication, speech, mandibular rest position, and others, are important aspects to assess during the diagnostic phase and considered while delivering dental interventions.

The closest speaking space is the minimum space found between teeth while the patient is speaking, with its name and definition being linked to Pound [1,2] and Silverman [3]. Its assessment is most frequently performed using speaking tests consisting of the patient saying the letter “S” or words containing it. Based on the current knowledge, the closest speaking space exhibits less variability than the freeway space [4] and is reproducible to about 1 to 2 mm [5] but with variations in the amount depending on the patient’s particularities, such as occlusion and skeletal features [2], and dental status, such as tooth wear [6]. The closest speaking space has clinical value, considering that oral rehabilitations, such as prosthetic ones, should be made with preservation of the closest speaking space in order to achieve an optimal functional outcome. Among its clinical applications, the closest speaking space is used to establish the vertical dimension of occlusion in edentulous patients [7,8] or establish the increase in the vertical dimension of occlusion, such as in patients with severe tooth wear [9]. In this regard, in edentulous patients, phonetic tests are used differently but often in conjunction to establish the functional vertical dimension of occlusion, i.e., tests with the letter “M” are used for determining it from the rest position, and tests with the letter “S” are used to assess its accuracy. In a study on this theme by Yamagata et al. [10], it was suggested that speech adaptation with an impact on the freeway space and the closest speaking space occurs due to different oral conditions, this being an aspect that needs to be further researched. Information is needed to establish the best parameters of these phonetic tests that provide the most accurate results.

Even if information on the closest speaking space is used nowadays in clinical practice, current evidence on it is scarce. Studies are performed using either clinical methods, which assess the interocclusal distance using interocclusal records [11] or the movement of the mandible during a phonetic test [10,12], or using instrumental methods, with the usage of the kineograph [13]. Digital analysis in dentistry in general, compared to physical analysis, frequently has the advantage of offering the possibility of an in-depth and more precise way of assessing the phenomenon. To the best of our knowledge, there are no reported studies using digital analysis of the closest speaking space in particular, nor other interocclusal spaces, the freeway space included, in general. The methods proposed in this research are new and have forward ways of improvement, but digital analysis of this aspect seems to be appropriate due to providing more precise and broader information on the closest speaking space, a theme that is important from a clinical point of view but supported by little scientific evidence.

The main study aim is to present some of the identified methods of digital analysis of the closest speaking space in dentate persons, exemplified by a case report. The secondary aim is to assess the particularities of the closest speaking space in dentate persons, following digital analysis of a case series.

## 2. Materials and Methods

This research received approval from The Scientific Research Ethics Commission of “Carol Davila” University of Medicine and Pharmacy, Bucharest (PO-35-F-03, No. 8366/2024). Study inclusion was confirmed after participants were informed about the research and signed an informed consent form to certify their agreement of participation.

**Participants.** This study was conducted on a convenience sample of adult participants with natural permanent dentition, all with occupations related to the dental field (dental students or dental technicians, with previous knowledge on dental procedures, phonetic tests included). Exclusion criteria included the following: impaired mandibular movement related to oral pain, temporomandibular disorders, or other factors; dental prosthetic restorations; coronal restorations with composite resin in the anterior teeth, fixed orthodontic appliances; previous allergic reactions to dental materials; severe speech defects.

**Data collection.** For all patients, traditional dental impressions using a universal tray and alginate were taken, then poured with Moldano dental gypsum (Kulzer, Hanau, Germany) to obtain maxillary and mandibular dental casts. Interocclusal records of the maximal intercuspal position and closest speaking space were created using polyvinyl siloxane (Occlufast, Zhermack, Badia Polesine, Italy). The same operator conducted this procedure for all patients included. The closest speaking space was recorded using a method based on the speaking test proposed by Silverman [12]. Participants were seated in an upright position without using a headrest; therefore, the head was unsupported. The impression material was placed on occlusal surfaces of the mandibular teeth. Afterwards, the participants, as previously instructed, pronounced the phonetic sound “S” and tried to maintain the corresponding mandibular position, without closing or moving the mandible, until the hardening of the material was complete.

Afterwards, dental casts were scanned using Swing DOF (DOF, Seoul, Republic of Korea) dental laboratory scanner, firstly in the position corresponding to maximal intercuspal position, and secondly in the position corresponding to closest speaking space.

The closest speaking space was digitally analyzed using the 3Shape 3D viewer software version 1.4. (3Shape) and occlusion map tool of 3Shape Ortho Analyzer (3Shape). Digital analysis followed the same two directions that are traditionally seen in clinical assessments of the closest speaking space, firstly by recording the absolute value of the closest speaking space, i.e., the minimum interocclusal distance between maxillary and mandibular teeth [11,13], and secondly by recording the amplitude of tooth movement during phonetic testing, representing the range of motion of the mandible for this specific situation, usually conducted by looking at the mandibular central incisors [12]. The different methods for digital analysis of the closest speaking space are presented below with the aid of a case report, followed by a presentation of the results of our analysis of a case series.

**Data analysis**. SPSS Statistics version 29 was used for data analysis. Group comparison was conducted using nonparametric test, i.e., Friedman and Wilkoxon test. Statistical significance was set at *p* < 0.05.

## 3. Results

### 3.1. Methods of Digital Assessment of Closest Speaking Space—Exemplification by a Case Report

A case report of a 26-year-old female patient is presented for the purpose of exemplifying the different methods for digital analysis of the closest speaking space.

#### 3.1.1. Digital Analysis of Closest Speaking Space Recorded by Interocclusal Distance

The first method for digital analysis involves uploading scans of the maxillary and mandibular casts placed in the position of the closest speaking space into the 3Shape 3D viewer.

**Figure 1:**
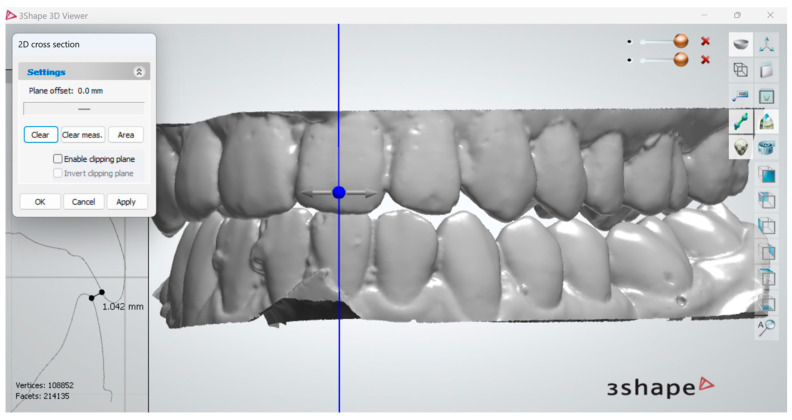
Measurement of interocclusal distance corresponding to closest speaking space at the level of the left lower central incisor, by the usage of 2D cross section of 3Shape 3D viewer.

**Figure 2:**
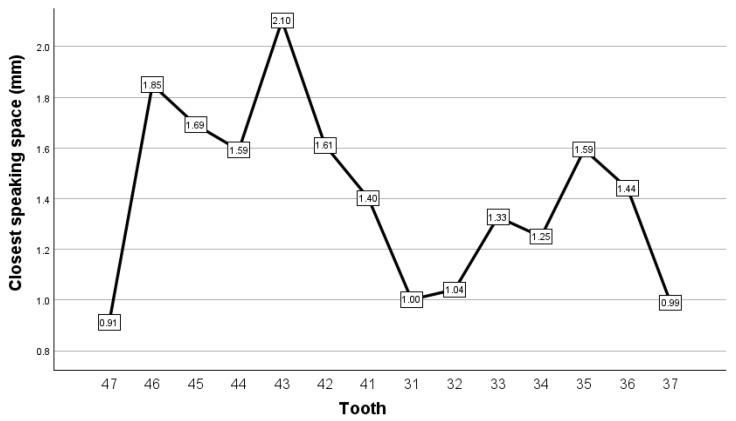
Closest speaking space, recorded as interocclusal distance, for the case reported for the purpose of method exemplification.

Measurements of interocclusal distance were conducted at the level of each mandibular tooth, with the aim of identifying the minimum interocclusal distance, it being recorded as the value of the closest speaking space at that tooth level. Measurements were taken using the 2D cross-section option in 3Shape 3D (Figure 1). The results registered for the closest speaking space are presented in Figure 2. In our view, digital analysis has the advantage of great precision of measurements, where such measurements can be easily taken digitally at the level of all antagonist teeth. Nonetheless, we found the process of identifying certain landmarks where the distance between antagonist teeth was lowest to be a relatively time-consuming process, which posed a certain degree of uncertainty in terms of accuracy.

**Figure 3:**
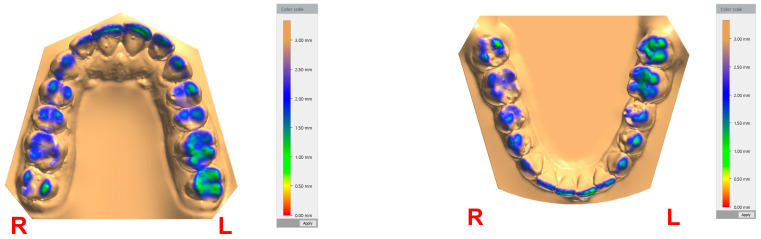
Occlusion maps corresponding to closest speaking space, generated by 3Shape Ortho Analyzer, for the case reported for the purpose of method exemplification.

The second method for digital analysis, which we identified, involves the use of occlusions maps generated by 3Shape Ortho Analyzer for the position corresponding to the closest speaking space (Figure 3). The closest speaking space can be easily compared between differently placed teeth using the color scale.

Our findings using each of the two methods identified above were similar in some regards. The closest speaking space ranged between approximately 1 and 2 mm for all of the patient’s teeth. The lowest values of the closest speaking space were observed in the left lower central incisor and in the most distally placed teeth, i.e., the second molars. A general trend of observing lower values of closest speaking was observed for the teeth placed on the left side compared to the ones placed on the right side, suggesting that a lateral mandibular movement to the left side occurred during the speaking test.

The two methods can be used together, as occlusion maps can be very helpful in identifying the landmarks where the closest speaking space has its lowest value, at the level of each tooth of the dental arch. In our view, the digital evaluation of the closest speaking space recorded by interocclusal distance can be used in both clinical practice and research and is, indeed, very appropriate for certain situations, e.g., to verify if changes to the closest speaking space occurred after a dental restorative treatment.

#### 3.1.2. Analysis of Closest Speaking Space, by Range of Motion of the Mandible During the Phonetic Test, Registered at the Level of All Teeth of the Dental Arch

Analyzing the closest speaking space by looking at the **amount of tooth movement** from the maximal intercuspation position to the position corresponding to the closest speaking space has two requirements: first, making digital scans for both positions, i.e., maximal intercuspal position and position corresponding to closest speaking space (1), and secondly, superimposing on one of the dental arches, on either maxillary or mandibular teeth (2). In this research, superimposition was conducted on the mandibular dental arch; therefore, the tooth movement was recorded using landmarks chosen on the maxillary teeth.

**Figure 4:**
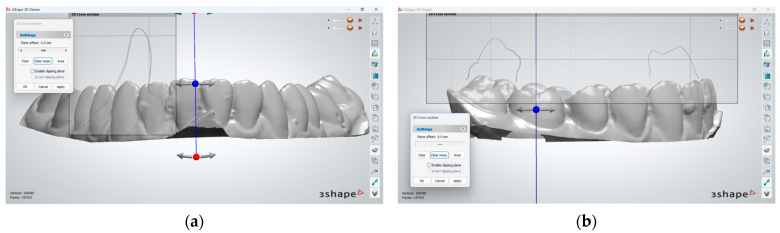
Superimposition of mandibular dental arches in the anterior (**a**) and posterior (**b**) area, for the case reported for the purpose of method exemplification.

To begin with, the superimposition of mandibular teeth for the two registered positions was assessed by visual inspection of the overlap at tooth level and was found to be acceptable in both anterior and posterior teeth (Figure 4). For all patients included in this study, when superimposition of the mandibular teeth for the two positions was found to be unsatisfactory, rescans were made until it was achieved.

**Figure 5:**
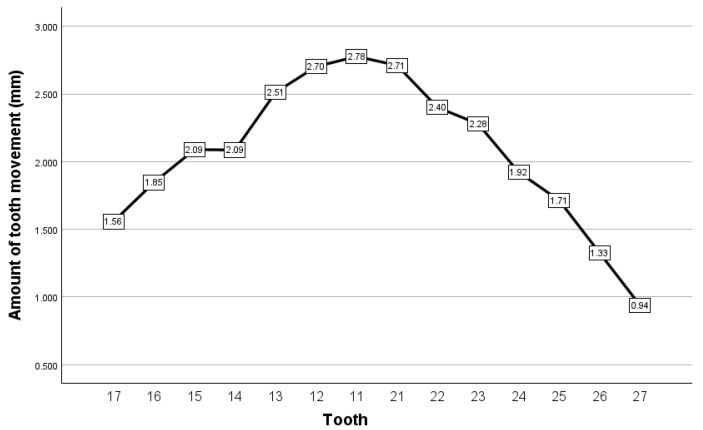
Closest speaking space, recorded as the amount of tooth movement from maximal intercuspal position, for the case reported for the purpose of method exemplification.

Afterwards, the amount of tooth movement was recorded at the level of each maxillary tooth. In order to do so, both maxillary casts were loaded (for both maximal intercuspation and closest speaking space), while mandibular casts were removed as they were regarded as unnecessary. We searched for an easily identifiable landmark on the maxillary tooth and then measured the distance between this landmark in both positions (maximal intercuspal position and position corresponding to closest speaking space). In this particular case, the results of our analysis were as follows: during the speaking test, tooth movement ranged from approximately 1 to 3 mm; on each side, the largest tooth movement was observed in the central incisors, and the amount of tooth movement decreased progressively as the tooth was placed more distally; left and right teeth registered different ranges of tooth movement, suggesting that some mandibular lateral movement to the left occurred during the speaking test (Figure 5). In our view, this method for analysis is easier to conduct than the previously mentioned method, whereby the interocclusal distance is recorded. This is due to the difficulties posed by the latter when it comes to identifying the exact location at the tooth level where the interocclusal distance is the lowest. However, our preferred method does require superimposition on one dental arch. Although this method does not generate the absolute value of closest speaking space (being minimum space between antagonistic teeth), it does provide valuable information on the mandibular movement during the speaking test, which is a factor that impacts the value of the interocclusal distance. This method can be used in further research on the closest speaking space and in research of other topics involving the assessment of mandibular movement and related outcomes for assessing the variation in the closest speaking space in patients with different skeletal classes or occlusal features.

**Figure 6:**
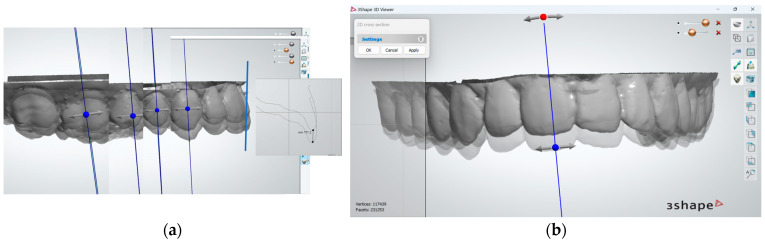
Analysis of tooth movement during the speaking test, in sagittal plane (**a**) and in frontal plane (**b**), for the case reported for the purpose of method exemplification.

**The direction of tooth movement** can be assessed in each tooth through digital analysis. The direction was previously recorded as the line drawn between the same tooth landmark in the two positions, i.e., maximal intercuspal position and position corresponding to closest speaking space. When interpreting the drawing, it is important to consider that the observed direction is opposite to the real one, as this research was conducted with superimposition on the mandibular dental arch. In this particular case, the results of our analysis of tooth movement were as follows. In the sagittal plane (Figure 6a), the direction of tooth movement varied, being more vertical for anterior teeth and increasingly oblique for more distally positioned teeth. Distal teeth registered an anterior tooth movement, which suggests that forward movement of the mandible occurred. Mandibular deviation to the left was identified when analyzing tooth movement in the frontal plane (Figure 6b), which is consistent with the results achieved through the other method of analysis, i.e., comparing the minimum interocclusal distance of the left and right placed teeth.

In our view, the two methods of analysis (analysis of occlusal distance and of tooth movement, respectively) provide complementary information, with consensus being observed on several aspects, e.g., on occurrence of mandibular deviation to the left but not on others, such as the value of closest speaking space.

### 3.2. Features of Closest Speaking Space by Digital Analysis—Case Series Presentation

#### 3.2.1. Participant’s Characteristics

The sample included eight participants, four males and four females, with ages ranging from 24 to 29 years, with a mean age of 24.9 years.

#### 3.2.2. Variation in Closest Speaking Space, Recorded as Interocclusal Distance, Across the Dental Arch

The value of the closest speaking space at the level of central incisors, where it is usually assessed, was variable, ranging from 0.86 to 3.95 mm for the mandibular right central incisor and from 0.98 to 3.82 for mm for the mandibular left central incisor.

**Figure 7:**
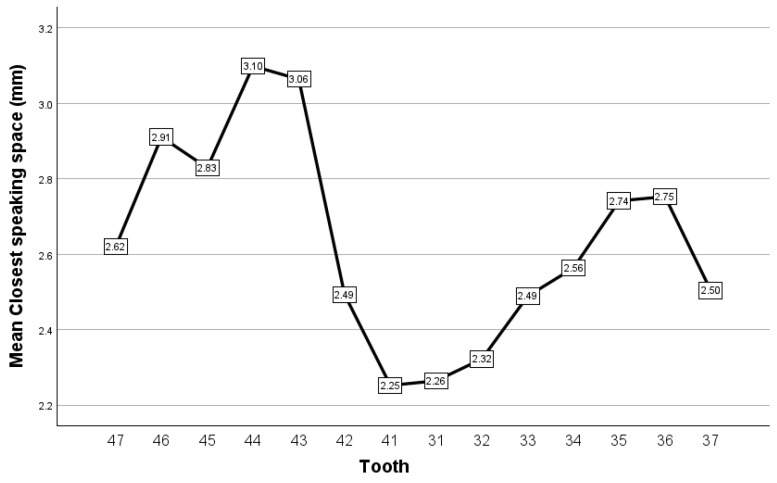
Closest speaking space, recorded as interocclusal distance, for the case series reported.

When assessed for variation across the dental arch, the pattern observed (Figure 7) was to register the smallest values of closest speaking space in the incisors, its value increasing as the teeth were placed more distally and then decreasing to reach its smallest values in posterior teeth, in the most distally placed ones, i.e., the second molars. The difference in value between the closest speaking space observed in the 14 analyzed teeth was statistically significant (Friedman test, *p* = 0.037), but none of the pairwise comparisons reached the level of statistical significance (significance values adjusted by the Bonferroni correction, *p* > 0.05 for all pairwise). As a general trend, the closest speaking space registered lower values for the left teeth compared to the right ones, which suggests the occurrence of mandibular deviation to the left; however, the difference in value of the closest speaking space was not statistically significant between right and left teeth (Wilcoxon test, *p* > 0.05 for all seven pairs of homologue teeth that were analyzed).

#### 3.2.3. Movement of the Mandible During Phonetic Test Used for Recording Closest Speaking Space, Assessed at Tooth Level, as Amount and Direction

**Figure 8:**
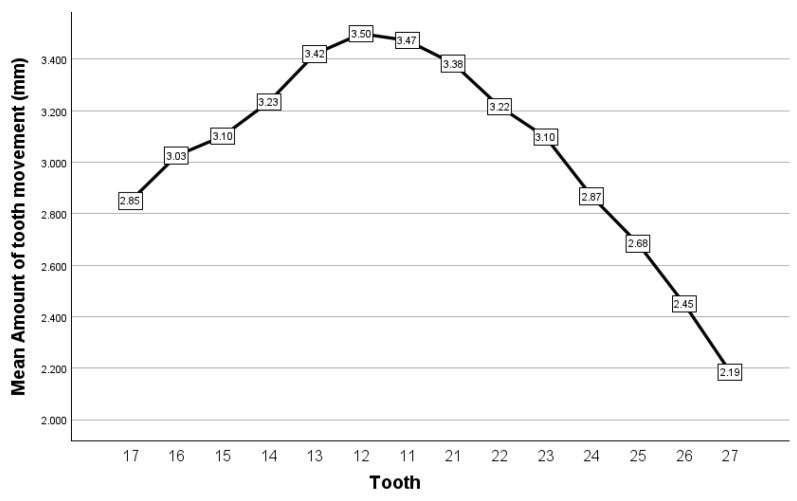
Closest speaking space, recorded as the amount of tooth movement from maximal intercuspal position, for the case series reported.

The amount of the tooth movement recorded at the level of the central incisors, where it is usually assessed, varied from 1.36 to 5.20 mm when assessed at the level of the maxillary right central incisor and from 1.33 to 5.16 mm when assessed at the level of the maxillary left central incisor. The amount of tooth movement had its highest values in the incisors and decreased progressively as the analyzed tooth was positioned more distally (Figure 8), the difference in value being statistically significant between the 14 analyzed teeth (Friedman test, *p* < 0.001). Through pairwise comparison, nine pairs of teeth had significance values adjusted by the Bonferroni correction *p* < 0.05, with a general trend of statistically significant differences being observed between left distal teeth and right anterior teeth (i.e., 27 and 21, 11, 12, 13; 26 and 11, 12 and 13; 25 and 11 and 12). In addition, as a general trend, the observed amount of tooth movement was lower for the left teeth compared to the right teeth, which suggests the occurrence of mandibular deviation to the left; however, when using the Wilcoxon test, the observed difference in value was statistically significant only for teeth 14–24, with the *p*-value being above 0.05 for the rest of the analyzed homologue teeth.

Analysis of tooth movement in the frontal plane revealed that, in this sample, a mandibular deviation to the left side occurred more frequently, being encountered in six out of eight cases. Analyzing these six cases, a statistically significant difference in tooth movement of all homologue teeth was observed, being lower in the left compared to the right teeth (Table 1).

## 4. Discussion

This study proposes methods for digital analysis of the closest speaking space that can also be applied for other functional spaces, such as freeway space. These methods of digital analysis can be used for both clinical practice and research. With regard to closest speaking space, the main findings of this research (while taking its limitations into account) are related to two aspects, which are important from a clinical point of view. Firstly, it is suggested that when oral rehabilitations are conducted (such as conventional or implant prosthetic rehabilitations), it is important to assess the preservation of the closest speaking space, not only in the central incisors but also in the most distally placed teeth, those being the instances where the closest speaking space registers its lowest values. Secondly, it is suggested that mandibular movement that occurs during phonetic testing should be better researched; our results show that both lateral and forward movement of the mandible occurs, which likely impacts the value of the closest speaking space recorded as the interocclusal distance.

When conducting dental treatments, obtaining a functional outcome is very important for the short- and long-term prognosis [14,15,16]. Existing means for obtaining information on oral functionality still present certain gaps and uncertainties but can be augmented using digital methods of analysis, with the latter promoting a more in-depth assessment compared to traditionally used physical methods. More so, gaining knowledge via digital assessment is increasingly feasible nowadays; its use has considerably changed dentistry in other areas of focus and registers an accelerated increase in use generally.

In this research, we proposed relatively simple methods of assessment of the closest speaking space that could be used for acquiring information, which is important from both a clinical and research point of view.

The first digital method proposed assesses the absolute value of closest speaking space recorded as the interocclusal space, by using impression material, as done in previous research [11,13]. The interocclusal distance can be measured more precisely digitally and can be easily achieved at the level of all teeth of the dental arch, ranging from the anterior to the posterior teeth. Nonetheless, difficulties arise in identifying the teeth landmarks required to register the true minimum value. The solution which was identified for this shortcoming is to use digitally generated occlusion maps for the position corresponding to the closest speaking space.

The second digital method proposed was based on the assessment of the tooth movement from the maximal intercuspal position to the position considered as being the one corresponding to the closest speaking space. In order to conduct this analysis, superimposition on one dental arch is required. Digital superimposition is increasingly used in dentistry to gain important clinical information [17], e.g., when assessing tooth movement during orthodontic treatment [18], or when assessing the occurrence and magnitude of tooth wear [19]. When compared to the previously mentioned applications, superimposition in this case is simpler, as no changes to dental arches occurred. Even so, it should be noted that, using this method, the actual closest speaking space (which is minimum interocclusal distance) is not assessed but, rather, the amplitude and direction of tooth movement between two positions during speaking tests. Even so, this component of analysis is important, considering that mandibular movement influences the value of the closest speaking space (the interocclusal distance) and that the pattern of mandibular movement during the speaking tests is related to the patient’s particularities, such as their occlusion and skeletal features [20,21].

The analyses, measurements, and superimpositions were conducted on 3D dental casts, the latter being an application previously used in research for different purposes [22]. Both digital analyses were conducted using 3Shape dental system software (3Shape 3D Viewer and 3Shape Ortho Analyzer) and could probably be conducted using other product variants. There are a range of applications which can be used for studying the range of mandibular movement [23], 3Shape included [24], but for the purposes of studying the closest speaking space, the options of recording and measuring the interocclusal distance should be available. In addition, the proposed method has the advantage of being relatively affordable and, therefore, promotes wider use. Several improvements in the proposed method could be tested, such as using an intraoral scanner for registering the position corresponding to the closest speaking space. Although digital analysis of the closest speaking space poses its own requirements and challenges, it seems to be a good alternative to existing physical methods used clinically. Shortcomings of the latter include aspects, such as lower precision of the measurement instruments used, occasional inaccuracy of measurement of interocclusal distance based on interocclusal records due to the consistency of the material used and sometimes due to voids, and difficulties in registering the amplitude of tooth movement for distally placed teeth and others. Such shortcomings can be overcome by using digital analysis, which is overall more precise and allows for a broader range of analysis.

The information on the closest speaking space gathered during our research confirms previous knowledge and raises new questions, which are important for clinical practice. As previous research suggests, values of the closest speaking space at the level of central incisors (where it is usually assessed) pose a certain degree of variability, which, in this research, range from approximately 1 to 4 mm. According to Pound [2], mentioned also in the Glossary of Prosthodontic terms [5], the value of the closest speaking space is usually around 1 to 2 mm. In previous research, values outside this range were found, with their variability being linked to patient features [11,20]. According to Sakar et al. [11], the closest speaking space shows larger values in dentate persons with Angle class II division 2 anomalies (mean = 2.66 mm; SD = 1.16 mm) and lower values in Angle class III anomalies (mean = 1.92 mm; SD = 0.95 mm). According to Souza et al. [20], the value of the closest speaking space is positively correlated with both vertical and horizontal overlap of the incisors. There are other factors (in addition to those already mentioned) that should be considered for having an impact on the closest speaking space, e.g., other dental occlusal particularities (whether static or dynamic), facial skeletal pattern, posture, and parafunctional habits. While the closest speaking space is variable between different individuals, it is constant and reproducible through phonetic tests in the same individual [25,26]. Therefore, in those instances in which the closest speaking space is clinically important to be assessed in dentate patients, a relatively simple way to do so is via digital analysis, as it can be conducted (if desired and considered clinically relevant) at the level of different teeth, as well as before or after treatment or in both moments in time, e.g., in the case of conventional or implant prosthetic rehabilitations. Additionally, considering the limited evidence on the topic, information on the reproducibility of phonetic tests acquired through physical analysis should be confirmed by digital analysis, considering the higher degree of precision of measurements involved in the latter.

Assessment of the closest speaking space is usually performed at the level of central incisors and recorded as the minimal occlusal distance at this level. By employing digital analysis, it was highlighted (as expected) that the closest speaking space registers different values at the level of different teeth in dentate persons. One important clinical aspect raised by this research, which should be confirmed by other research studies, is that the lower values of the closest speaking space are encountered at the level of both the most anterior and most distally placed teeth, with similar values sometimes being recorded. This information suggests that when prosthetic restorations are made in the most distally placed teeth, the value of the closest speaking space should be assessed at that level; this is especially advisable in patients whose closest speaking space has lower values, as in class III patients [11].

The values of the observed interocclusal distance (corresponding to closest speaking space) are related to mandibular movement that occurs during the speaking test. Digital analysis conducted during this research reveals that such movement was, as expected, not symmetrical, with a mandibular deviation to the left side occurring most frequently. These findings contradict the study of Zhang et al. [27], which concluded that almost no deviation of the mandible is observed in the left or right direction during phonetic tests, when measurement is conducted using physical methods, with a vernier caliper. This contradiction is unlikely to be explained by a complete absence of lateral deviation of the mandible, as the odds of achieving a perfectly symmetrical movement are very low. Instead, it is suggested that the most probable explanation lies with the difficulty of assessment and recording of the mandibular movement using physical methods, as physical methods have lower precision. This is particularly important in this case, when considering the very low range of mandibular movement. Previous evidence shows that patients with certain features, such as facial skeletal class, are associated with a different pattern of lateral mandibular movement [21]. In such instances, the amplitude of the mandibular movements, as observed during phonetic testing, should be further investigated to identify those patients in which it is more frequently encountered. In addition, aspects related to the impact of these mandibular movements on the interocclusal distance of the closest speaking space and the extent to which it is clinically relevant should be assessed. Finally, the results of this research suggest that, during phonetic testing, a forward movement of the mandible occurs. This aspect is in accordance with a study conducted by Zhang et al. [27], which found that during speech, the mandible was more forward compared to the maximal intercuspal position. Pound [28] also highlighted the importance of acknowledging the degree of forward movement of the mandible, which impacts the value of closest speaking space, especially in the posterior teeth, depending on occlusion particularities. These ideas are supported by the findings of subsequent research studies [11,20]. Therefore, it is suggested that digital analysis of mandibular movement during speech is advisable in individual cases, as information on mandibular movement has been previously shown to contribute to a better functional integration of the prosthetic restorations [24,29].

This research has several limitations. Among these, we note that there are other digital applications that could be used and should be tested in terms of suitability as a method for studying the closest speaking space; this research highlights certain main features that should be included in them. As a future direction of improvement, intraoral scanning of the position corresponding to the closest speaking space could be used. Other limitations could be linked to measurement or registration errors, the small number of participants, and registration of the closest speaking space through a speaking test. Future research on larger sample sizes is recommended to confirm the information found on this topic.

## 5. Conclusions

This research proposes new digital methods for the analysis of the closest speaking space. The proposed digital methods display a range of advantages, including the possibility of a more precise and in-depth analysis when compared to conventional physical methods, the possibility of a broader range of application (e.g., for the study of other functional spaces, such as freeway space, and the study of mandibular movements), the likelihood of both clinical and research applications, all while representing an operationally accessible, low-risk, and low-cost method. Important clinical information generated by this research with regard to the closest speaking space in dentate persons, the lowest values of interocclusal distance found in most anterior and most distal teeth, and the lateral and forward movement of the mandible during speaking tests should be confirmed by future research using larger sample sizes.

## Figures and Tables

**Table 1:** Amount of tooth movement in right vs. left teeth, for the participants with mandibular deviation to the left side.

Homologue Teeth	Right Tooth Movement Mean (Minimum; Maximum)	Left Tooth Movement Mean (Minimum; Maximum)	*p*
17–27	2.89 (1.19; 4.53)	2.23 (0.72; 4.02)	0.028
16–26	3.09 (1.26; 4.70)	2.52 (0.86; 4.12)	0.043
15–25	3.19 (1.29; 4.60)	2.79 (0.98; 4.47)	0.043
14–24	3.37 (1.42; 5.05)	2.99 (1.10; 4.75)	0.028
13–23	3.56 (1.43; 5.18)	3.26 (1.17; 5.07)	0.046
12–22	3.67 (1.39; 5.20)	3.40 (1.22; 5.05)	0.028
11–12	3.64 (1.36; 5.16)	3.56 (1.33; 5.16)	0.028
Wilcoxon test			

## Data Availability

The original contributions presented in this study are included in the article; further inquiries can be directed to the corresponding author.
